# Efficacy and Safety of Cadonilimab in Digestive System Neoplasms: A Systematic Review and Single-Arm Meta-Analysis

**DOI:** 10.3390/ph19091348

**Published:** 2026-08-26

**Authors:** Qiuqi Zhuang, Zhitao Yang, Yan Liu

**Affiliations:** Academy of Integrative Medicine, Fujian University of Traditional Chinese Medicine, Fuzhou 350108, China

**Keywords:** cadonilimab, digestive system neoplasms, efficacy, safety, systematic review, meta-analysis

## Abstract

**Objective:** This study aimed to systematically evaluate the efficacy and safety of cadonilimab in the treatment of digestive system neoplasms. **Methods:** PubMed, Embase, Cochrane Library, Web of Science, Wiley Online Library, CNKI, Wanfang Data, CBM, and VIP databases were systematically searched to collect randomized controlled trials, non-randomized studies, and real-world studies investigating cadonilimab for the treatment of digestive system neoplasms. The initial search spanned from the inception of the databases to 4 February 2026, with an update search conducted in June 2026. Meta-analysis was performed using Stata 16.0 to systematically assess efficacy and safety outcomes. **Results:** A total of 10 studies, predominantly single-arm or retrospective, were included in this meta-analysis. Regarding the primary outcomes, because of the clinical heterogeneity across tumor types, pooled estimates should be interpreted as a summary across diverse diseases rather than as a single-tumor estimate. The overall pooled disease control rate (DCR) was 84% (95% CI = 76–91%), with tumor-specific DCRs of 87% in ESCC, 79% in HCC, 88% in G/GEJ adenocarcinoma, and 86% in PDAC. The pooled incidence of any-grade treatment-related adverse events (TRAEs) was 98% (95% CI = 95–100%), and the pooled incidence of any-grade immune-related adverse events (irAEs) was 32% (95% CI = 18–47%). For secondary outcomes: The partial response (PR) rate was 33% (95% CI = 21–46%), and the progressive disease (PD) rate was 11% (95% CI = 5–18%). The pooled incidence of grade ≥ 3 TRAEs was 52% (95% CI = 38–66%), and the pooled incidence of grade ≥ 3 irAEs was 11% (95% CI = 8–16%). However, incomplete reporting of low-frequency AEs across studies likely underestimates the pooled irAE rate. **Conclusions:** This study suggests that cadonilimab has the potential for antitumor activity in digestive system neoplasms. The observed safety profile was consistent with the known toxicities of immunotherapy, with notably high rates of TRAEs warranting vigilant monitoring. These findings should be considered hypothesis-generating and are insufficient to establish clinical benefit.

## 1. Introduction

Globally, digestive system neoplasms, including gastric, liver, esophageal, and pancreatic cancers, are characterized by high incidence and mortality rates and a dismal long-term prognosis [1]. These malignancies account for over a quarter of all cancer cases and a third of cancer-related deaths worldwide, imposing a substantial socioeconomic burden [2]. Patients with esophageal squamous cell carcinoma (ESCC) may be asymptomatic early, but as the disease advances locally, they typically develop progressive dysphagia: initially difficulty swallowing solids, later progressing to liquids due to tumor-induced esophageal narrowing [3]. The management of gastric/gastroesophageal junction (G/GEJ) adenocarcinoma is guided by specific biomarkers. Currently, HER2 positivity, high microsatellite instability (MSI-H), and programmed death ligand 1 (PD-L1) expression are established predictive biomarkers for certain therapies in advanced G/GEJ cancers [4,5]. Although direct head-to-head comparisons are lacking, anti-PD-1 and anti-PD-L1 agents are often regarded as clinically equivalent. CTLA-4 inhibitors have also been evaluated in gastroesophageal cancer but have not demonstrated significant efficacy in the advanced setting [4]. Hepatocellular carcinoma (HCC) usually develops through a continuous progression from chronic liver disease to cirrhosis, the most critical precancerous condition. Cirrhosis most commonly arises from chronic viral hepatitis, alcoholic steatohepatitis, or non-alcoholic steatohepatitis; persistent liver injury ultimately leads to liver cancer [6]. Pancreatic ductal adenocarcinoma (PDAC) is a highly aggressive digestive system neoplasm. Its mortality rate ranks among the highest of all solid organ cancers, and the 5-year survival rate remains below 10%, a figure that has barely improved over decades, reflecting an extremely poor prognosis [7].

The rapid advancements in cancer immunology and molecular biology have significantly reshaped the therapeutic landscape for digestive system neoplasms. According to the NCCN Clinical Practice Guidelines, immune checkpoint inhibitors have become part of the standard first-line treatment for certain digestive system neoplasms. They are recommended in combination with chemotherapy for G/GEJ cancer [8], with anti-angiogenic agents or targeted therapies for hepatocellular carcinoma [9], and, in pancreatic cancer, are primarily limited to later-line treatment for patients with specific molecular subtypes (e.g., MSI-H/dMMR) [10]. However, even within this subgroup, some patients do not respond to treatment and fail to achieve adequate tumor control. Therefore, effective and innovative therapeutic strategies for digestive system neoplasms are urgently needed.

With advances in protein engineering, bispecific antibodies have shown therapeutic potential through dual-targeting mechanisms and represent a novel direction in oncology [11]. Cadonilimab, a bispecific antibody targeting PD-1 and CTLA-4, simultaneously engages these two critical immune pathways to exert synergistic antitumor effects [12,13]. Compared with conventional PD-1 plus CTLA-4 inhibitor combinations, cadonilimab features an Fc-null design that abolishes Fcγ receptor and C1q binding, potentially reducing lymphocyte depletion and cytokine release. Its symmetric tetravalent structure further provides a unique basis for enhanced antitumor activity and safety [12,13,14]. Preliminary clinical evidence of antitumor activity has emerged from the phase Ib/II COMPASSION-03 study [15]. However, current clinical studies of cadonilimab in digestive system neoplasms are mostly small, single-arm trials with outcomes that vary considerably across settings. This fragmented evidence challenges clinical decision-making, and high-quality data remain insufficient. A systematic synthesis of all available evidence is therefore needed to comprehensively assess the efficacy and safety of this agent. Accordingly, we conducted a meta-analysis to pool the existing efficacy and safety data, aiming to provide a descriptive summary that may inform clinical practice and guide future research.

## 2. Methods

This study was conducted in strict accordance with the Preferred Reporting Items for Systematic Reviews and Meta-Analyses (PRISMA) guidelines. The study protocol was registered with the PROSPERO international prospective register of systematic reviews (registration ID: CRD420261300920).

### 2.1. Search Strategy

This study systematically searched nine electronic databases, including PubMed, Embase, Cochrane Library, Web of Science, Wiley Online Library, China National Knowledge Infrastructure (CNKI), Wanfang Data, China Biology Medicine (CBM), and VIP, supplemented by a manual search of grey literature. We aimed to collect randomized controlled trials, non-randomized studies, and real-world studies on cadonilimab for the treatment of digestive system neoplasms. The initial search spanned from the inception of the databases to 4 February 2026, with an update search conducted in June 2026. Search terms included core concepts such as “Cadonilimab”, “AK104”, and “Digestive System Neoplasms”, combined using Boolean operators “AND” and “OR”. The search strategy was flexibly adjusted according to the characteristics and requirements of each database. The complete search strategies for all databases are provided in Supplementary File S1.

### 2.2. Eligibility Criteria

In this review, no restrictions were imposed on language or sample size, and only complete, peer-reviewed, published studies were considered. Studies meeting the following criteria were included in this meta-analysis: (a) Patients with a confirmed diagnosis of digestive system neoplasms, regardless of disease stage, prior treatment history, geographic region, or ethnicity, were included. (b) Patients received cadonilimab as monotherapy or in combination with other regimens (e.g., chemotherapy or other active agents). (c) At least one efficacy outcome or safety outcome was reported. Studies meeting any of the following criteria were excluded: (a) Key outcome data were missing or unavailable. (b) Publication type was systematic reviews and meta-analyses, case reports, preclinical studies, editorials, or conference proceedings. (c) Full text was unavailable, or the study was a duplicate publication.

### 2.3. Data Extraction

Two investigators independently performed the literature screening and data extraction. First, retrieved records were imported into EndNote 21 software for duplicate removal. Subsequently, preliminary screening and full-text review were conducted based on predefined inclusion and exclusion criteria. Finally, eligible studies were selected for data extraction. The extracted data included the first author, publication year, study design, tumor type, sample size, median age, combination regimen, efficacy outcomes, and safety outcomes. For studies with a comparator arm, data from the eligible cadonilimab-containing arm were extracted and pooled with those from single-arm studies. Disagreements were resolved through discussion; when consensus could not be reached, a third senior investigator adjudicated. In cases of missing data, corresponding authors were contacted to obtain the information. The primary outcome measures were disease control rate (DCR), any-grade treatment-related adverse events (TRAEs), and any-grade immune-related adverse events (irAEs). Secondary outcome measures included partial response (PR) rate, progressive disease (PD) rate, grade ≥ 3 TRAEs, grade ≥ 3 irAEs, and specific categories of adverse events (including hematologic toxicity, gastrointestinal toxicity, and hepatic toxicity).

### 2.4. Quality Assessment

The Jadad scale was used to assess the quality of randomized controlled trials, evaluating random sequence generation, allocation concealment, blinding, and withdrawals and dropouts. The total possible score was 7, with scores of 1–3 considered low quality and 4–7 considered high quality. The methodological index for non-randomized studies (MINORS) was employed for quality assessment. For non-comparative single-arm studies, the first eight items were used (maximum score of 16). For comparative studies, all 12 items were applied (maximum score of 24). Each item was scored from 0 to 2, where 0 indicated not reported, 1 indicated inadequately reported, and 2 indicated adequately reported.

### 2.5. Statistical Analysis

Stata software (version 16.0) was used for the meta-analysis of single-arm proportions. Event rates from multiple studies were pooled and expressed as proportions with 95% confidence intervals (CIs). Heterogeneity was assessed using the Q test and the I^2^ statistic. Because some event rates approached 0 or 1, which would produce invalid forest plots with the conventional metan command, the metaprop command was applied with the Freeman–Tukey double arcsine transformation. Given the varying degrees of heterogeneity across subgroups, all pooled estimates were calculated using random-effects models. Sensitivity analyses were performed by omitting individual studies one by one to identify potential sources of heterogeneity. Prespecified subgroup analyses based on tumor type, study design, study scope, and combination regimen were also conducted. Publication bias was evaluated using funnel plots and further assessed with Egger’s regression test. A two-sided *p*-value < 0.05 was considered statistically significant.

## 3. Results

### 3.1. Study Selection

**Figure 1:**
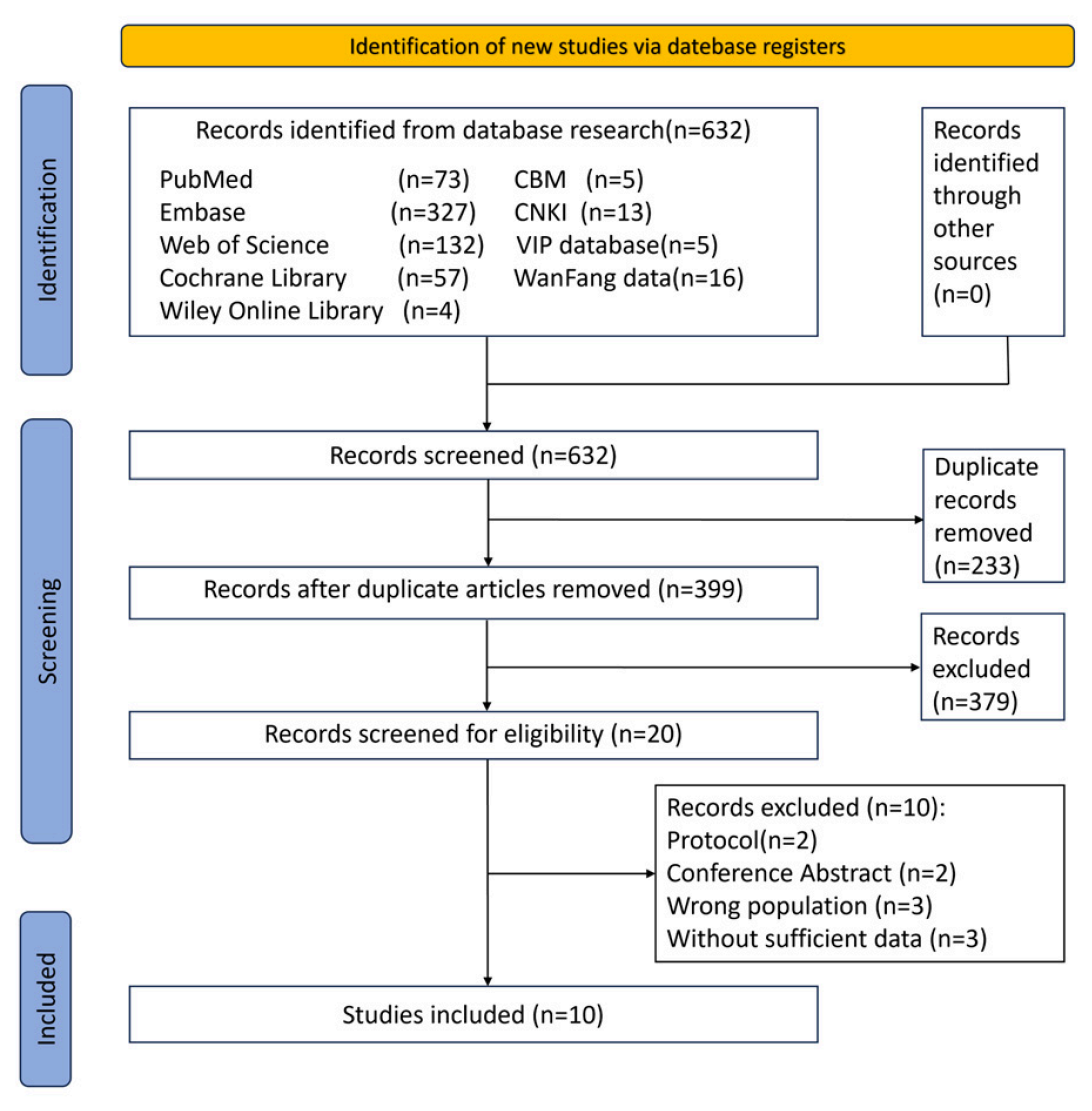
PRISMA flow diagram of study selection.

A total of 632 records were initially identified through database searches. After removing 233 duplicate studies, the remaining records were screened step by step based on the inclusion and exclusion criteria. Ultimately, 10 studies were included in the analysis [15,16,17,18,19,20,21,22,23,24], comprising 1 randomized controlled trial [20], 5 single-arm clinical trials [15,16,18,21,22], and 4 real-world retrospective studies [17,19,23,24]. The literature screening process is depicted in Figure 1.

### 3.2. Study Characteristics

The sample sizes of the included studies ranged from 14 to 305 patients. DCR was defined as the proportion of patients achieving a best overall response of CR, PR, or SD according to Response Evaluation Criteria in Solid Tumors (RECIST) version 1.1, which was uniformly applied for efficacy assessment across all included studies. Four studies were single-center, and six were multicenter. A total of 11 eligible arms were included in this meta-analysis (Gao et al., 2023, [15] contributed separate ESCC and HCC cohorts). Given that all included cohorts were derived from independent large-scale research institutions and clinical programs, the possibility of overlapping patient populations was considered low. All included studies were published within the last three years. The included studies were of moderate to high quality, with the main methodological deficiencies being the lack of prospective sample size calculation and inadequate reporting of loss to follow-up in some studies. The basic characteristics and quality assessment results of the included studies are presented in Table 1.

### 3.3. Primary Outcomes

**Figure 2:**
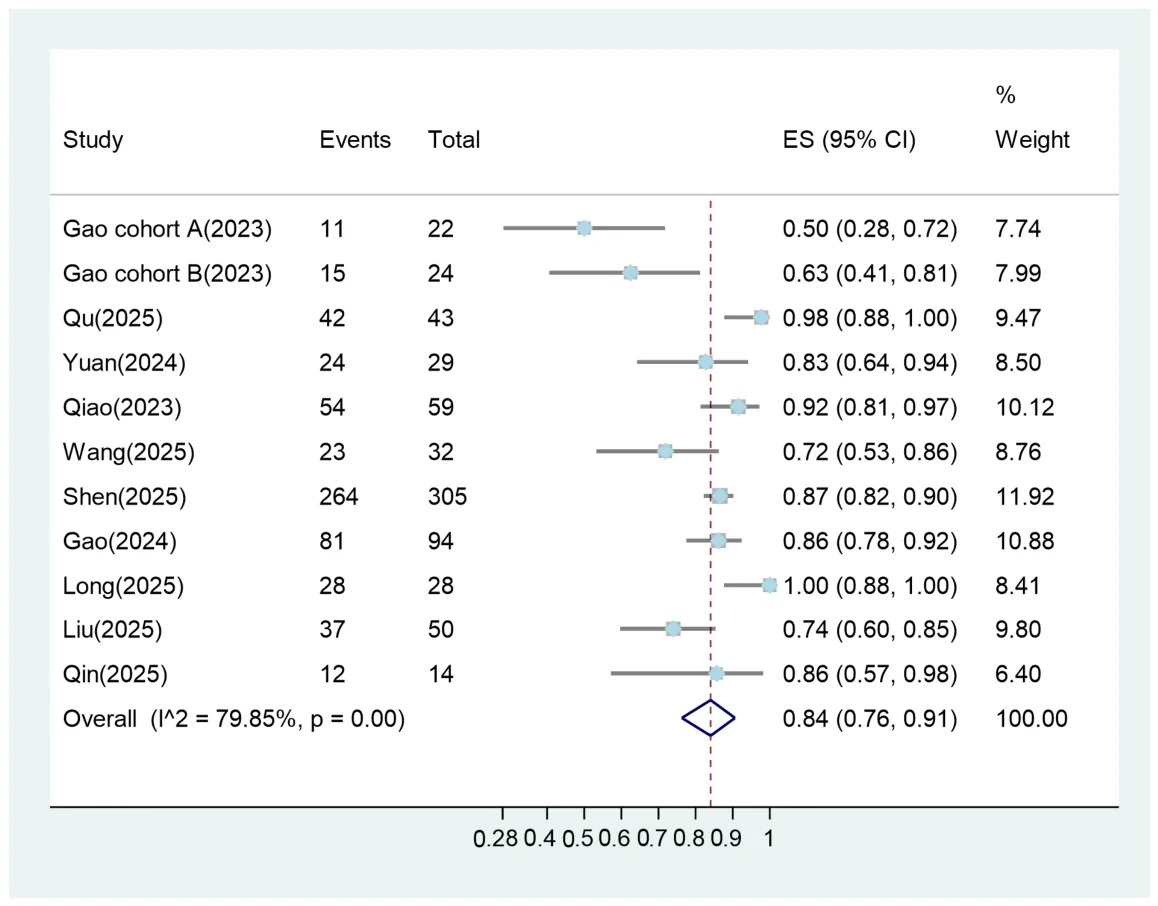
Forest plot of results for DCR.

**Figure 3:**
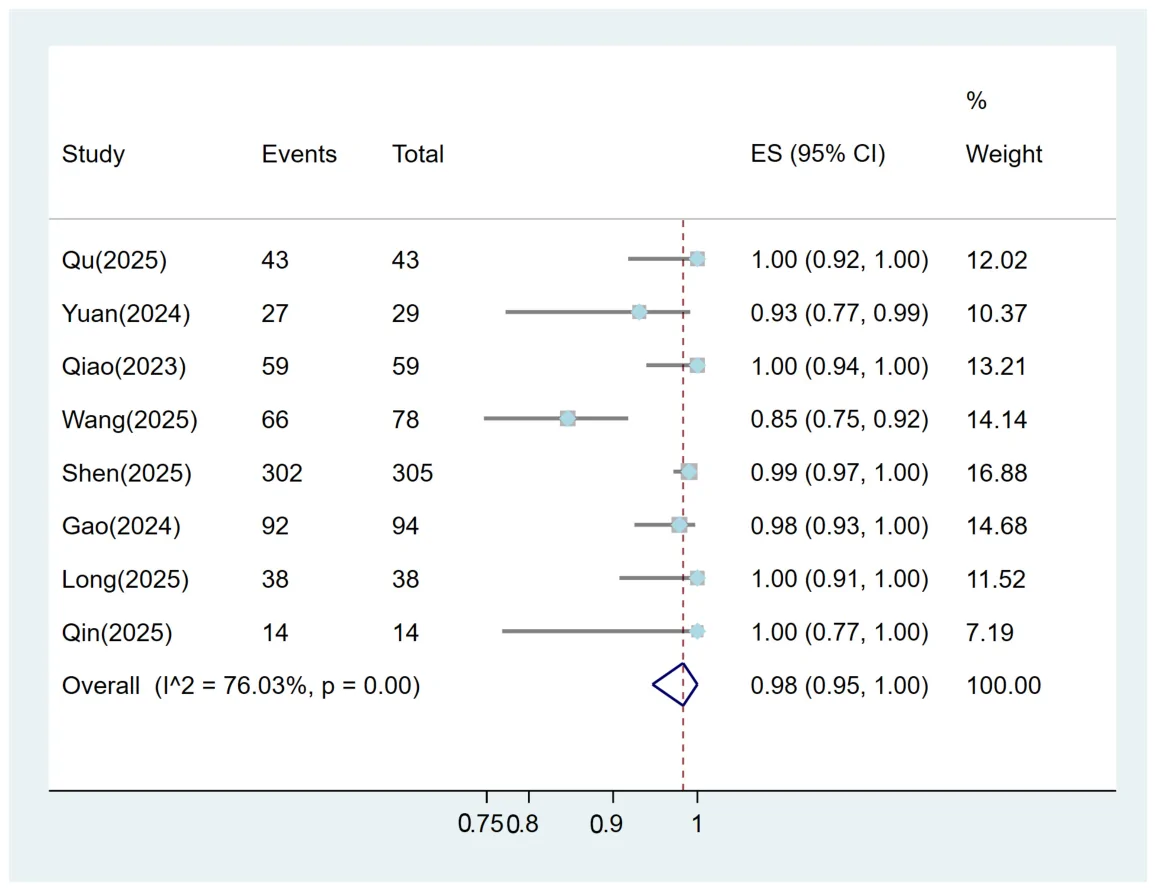
Forest plot of results for any-grade TRAEs.

**Figure 4:**
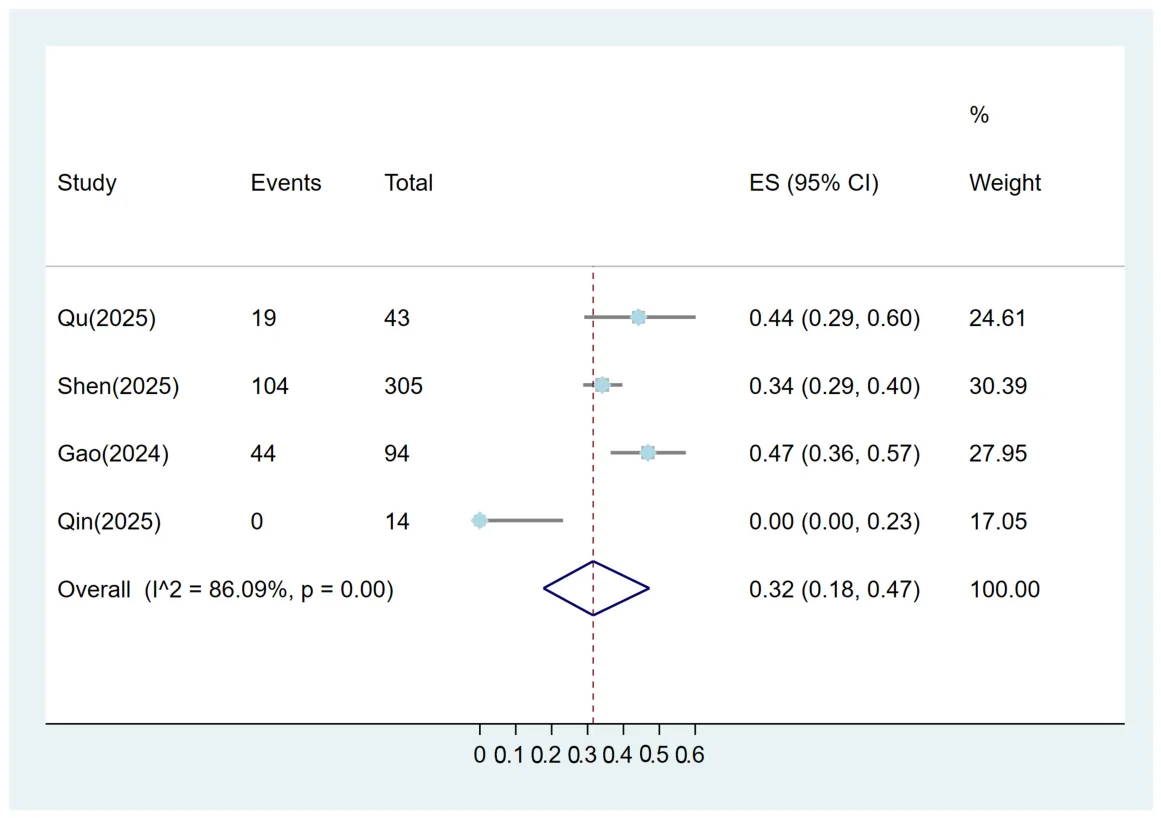
Forest plot of results for any-grade irAEs.

For efficacy, all included studies [15,16,17,18,19,20,21,22,23,24] reported DCR. Meta-analysis results showed that the pooled DCR for patients with digestive system neoplasms treated with cadonilimab was 84% (95% CI = 76–91%; Figure 2). Regarding safety, eight studies [16,17,18,19,20,21,22,24] reported any-grade TRAEs, and the pooled incidence was 98% (95% CI = 95–100%; Figure 3). Four studies [16,20,21,24] reported any-grade irAEs, and the pooled incidence was 32% (95% CI = 18–47%; Figure 4). The results of the heterogeneity test indicated the presence of heterogeneity across outcomes (I^2^ > 50%, *p* < 0.01).

### 3.4. Secondary Outcomes

For efficacy outcomes, nine studies reported PR, with a pooled rate of 33% (95% CI = 21–46%). Eight studies reported PD, with a pooled rate of 11% (95% CI = 5–18%). For safety outcomes, eight studies reported grade ≥ 3 TRAEs, with a pooled incidence of 52% (95% CI = 38–66%). Four studies reported grade ≥ 3 irAEs, with a pooled incidence of 11% (95% CI = 8–16%). Forest plots for PR and PD are presented in Figures S1 and S2; forest plots for grade ≥ 3 TRAEs and grade ≥ 3 irAEs are presented in Figures S3 and S4.

All included studies reported specific categories of adverse events, including hematologic toxicity, gastrointestinal toxicity, and hepatic toxicity. Hematologic toxicity included thrombocytopenia, neutropenia, leukopenia, and anemia. Gastrointestinal toxicity included decreased appetite, weight loss, diarrhea, and vomiting. Hepatic toxicity included elevated aspartate aminotransferase (AST), elevated alanine aminotransferase (ALT), hyperbilirubinemia, and hypoalbuminemia. The pooled results of the meta-analysis for these specific adverse events are presented in Table 2.

### 3.5. Subgroup Analysis

Due to the observed heterogeneity among studies, subgroup analyses were further conducted based on specific types of digestive system neoplasms, study design, study scope, and combination regimen to investigate potential sources of clinical heterogeneity. The results of the subgroup analysis for DCR are presented in Table 3, and those for any-grade TRAEs are presented in Table 4.

### 3.6. Sensitivity Analysis

**Figure 5:**
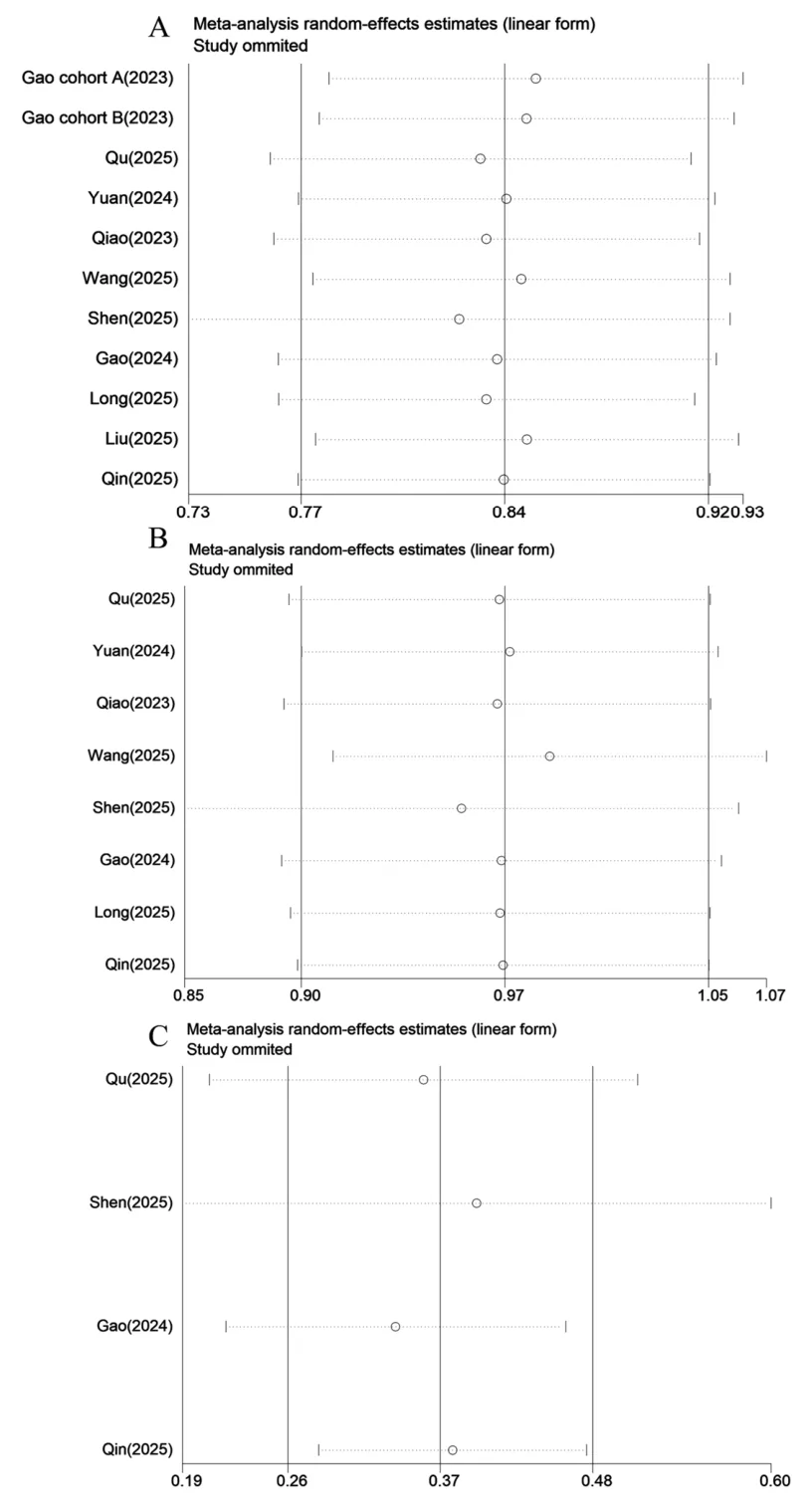
Sensitivity analysis of the primary outcomes: DCR (**A**), any-grade TRAEs (**B**), and any-grade irAEs (**C**).

Sensitivity analysis was performed by sequentially omitting individual studies to exclude the disproportionate influence of any single study on the overall meta-analysis results, and the findings for the primary outcomes were presented. After excluding cohort A in the study by Gao (2023) [15], heterogeneity in the DCR group was reduced (I^2^ = 74.66%, *p* < 0.01), and the pooled rate increased to 86% (95% CI = 80–92%). After excluding the study by Wang (2025) [19], heterogeneity in the any-grade TRAEs group was eliminated (I^2^ = 0.00%, *p* = 0.46), and the pooled rate was 100% (95% CI = 99–100%). After excluding the study by Qin (2025) [24], heterogeneity in the any-grade irAEs group was reduced (I^2^ = 64.96%, *p* = 0.06), and the pooled rate was 40% (95% CI = 31–50%). These results are shown in Figure 5.

### 3.7. Bias Assessment

**Figure 6:**
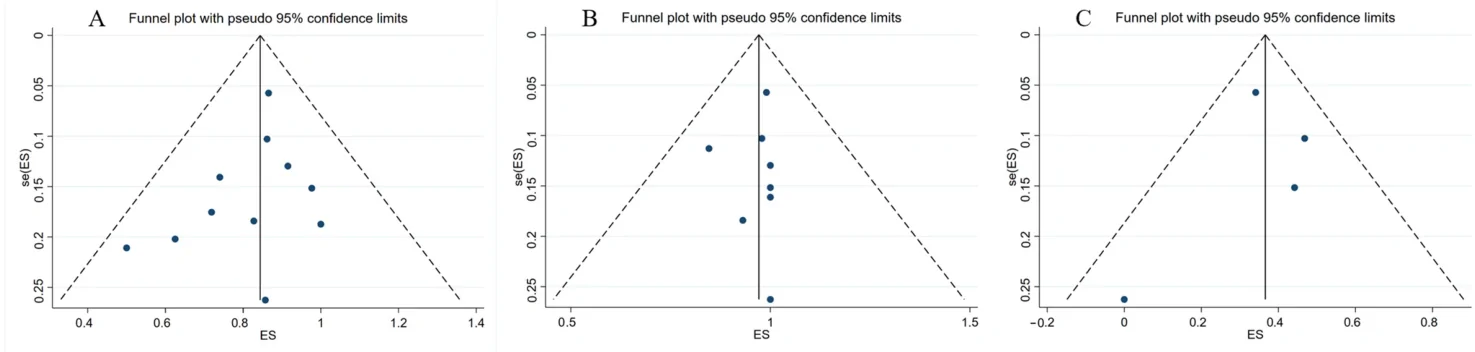
Funnel plots for the primary outcomes: DCR (**A**), any-grade TRAEs (**B**), and any-grade irAEs (**C**).

Funnel plots were constructed for the primary outcome measures to evaluate the risk of publication bias. Visual inspection revealed generally symmetrical plots. Further assessment using Egger’s regression test yielded *p*-values > 0.05 for all primary outcomes (DCR: *p* = 0.280; any-grade TRAEs: *p* = 0.728; any-grade irAEs: *p* = 0.807), as shown in Figure 6. Nevertheless, the small number of included studies limits the statistical power of these tests and precludes a reliable assessment of publication bias, necessitating cautious interpretation of these negative findings.

## 4. Discussion

In East Asia, the incidence and mortality of digestive system neoplasms, particularly gastric, liver, and esophageal cancers, are the highest worldwide and continue to rise. By 2040, new cases and deaths are projected to increase by 58% and 73%, respectively [2]. Bispecific antibodies have substantially advanced cancer immunotherapy by enabling novel mechanisms of action and therapeutic applications not possible with conventional IgG-based antibodies [25]. Cadonilimab (AK104) is a PD-1/CTLA-4 bispecific antibody developed by Akeso [12]. In 2022, it was approved in China for recurrent or metastatic cervical cancer whose disease progressed during or after platinum-based chemotherapy. A meta-analysis reported a pooled DCR of 81.8% in cervical cancer [26]. Cadonilimab has also attracted considerable attention for its potential use in digestive system neoplasms, but studies in this field remain predominantly small, single-arm, and exploratory. Consequently, efficacy and safety data are limited, and clinical practice faces challenges, including off-label use without evidence-based support. This study systematically evaluates the efficacy and safety of cadonilimab in esophageal cancer, gastric/gastroesophageal junction adenocarcinoma, hepatocellular carcinoma, and pancreatic ductal adenocarcinoma through a meta-analysis. By pooling all currently available data, this study aims to provide a descriptive summary to inform clinical decision-making and guide future research.

Across the 11 cohorts from 10 studies included in this review, the estimated DCR varied by tumor type: 87% in ESCC, 79% in HCC, 88% in G/GEJ adenocarcinoma, and 86% in PDAC. As an exploratory summary across these biologically distinct cancer types, the overall pooled DCR was 84%, with a PD rate of 11%. These rates indicate that a substantial proportion of patients achieved disease control or better, while disease progression occurred in a minority of cases. However, given the single-arm nature of the included studies, these findings cannot be attributed solely to the therapeutic effect of cadonilimab. These four tumor types present distinct immunotherapy challenges. In advanced ESCC, first-line PD-1 inhibitors plus chemotherapy are standard, while adding CTLA-4 inhibition is limited by safety concerns [27]. In advanced HCC, PD-1/PD-L1 inhibitors combined with anti-angiogenic agents or CTLA-4 inhibitors are now preferred, given that monotherapy achieves only a 15–20% objective response rate and 30% of patients exhibit resistance [28]. In advanced G/GEJ cancer, PD-1/PD-L1 inhibitor monotherapy provides only modest benefit [29]. Pancreatic cancer, with its highly immunosuppressive microenvironment, has responded poorly to immunotherapies, including checkpoint inhibitor monotherapy, underscoring an urgent need for novel strategies [30]. Cadonilimab, a symmetric tetravalent bispecific antibody with an Fc-null design, blocks PD-1 binding to PD-L1 and PD-L2, as well as CTLA-4 binding to B7-1 and B7-2 [12,13]. Bispecific antibodies target two distinct antigens, enabling mechanisms unattainable with monospecific antibodies [14]. Cadonilimab shows activity comparable to dual CTLA-4/PD-1 blockade and exhibits enhanced binding avidity where PD-1 and CTLA-4 are co-expressed at high density, a property absent from monospecific anti-PD-1 antibodies [13]. PD-1 and CTLA-4 drive T-cell exhaustion through non-redundant pathways, and PD-1^+^CTLA-4^+^ double-positive CD8^+^ T cells represent the most severely dysfunctional subset in the tumor microenvironment. Dual blockade synergistically restores their proliferation and effector function while attenuating the suppressive activity of intratumoral regulatory T cells, reshaping the immune microenvironment [31,32]. The Fc-null design abolishes FcγR and C1q binding, thereby reducing lymphocyte depletion and macrophage-driven cytokine release, which may enable more precise targeting of tumor-infiltrating lymphocytes [12].

Prior immune checkpoint inhibitor (ICI) exposure can reactivate autoreactive and tumor-specific T cells, causing irAEs in up to 70% of patients, depending on cancer type and regimen [33]. In our study, any-grade TRAEs occurred in most patients receiving cadonilimab, yet the incidence of any-grade irAEs was 32%, and that of grade ≥ 3 irAEs was only 11%. This safety profile aligns with the expected toxicity burden of dual-checkpoint blockade in advanced cancers, although the clinical significance of these findings requires further study. This safety characteristic is thought to be related to its Fc-null design, which eliminates FcγR-mediated effector functions (such as ADCC and CDC) and associated pro-inflammatory cytokine release, thereby potentially reducing the risk of irAEs. Furthermore, its bispecific structure enhances target selectivity, providing a mechanistic basis for improving safety while enhancing efficacy [34]. Specific categories of adverse events were also pooled and analyzed in this study. Pooled incidences of hematologic toxicities were 29% for thrombocytopenia, 42% for neutropenia, 43% for leukopenia, and 35% for anemia. For grade ≥ II hematologic toxicity during ICI therapy, management typically includes withholding immunotherapy and administering corticosteroids or intravenous immunoglobulin (IVIG) based on toxicity type and severity [35]. Treatment decisions should be guided by clinical bleeding symptoms rather than laboratory platelet counts alone [35]. For cadonilimab, permanent discontinuation is recommended for all grade 4 and selected grade 3 hematologic toxicities, any recurrent grade 3 toxicity, or grade 2/3 toxicity persisting beyond 12 weeks after the last dose, as well as when corticosteroids cannot be tapered to ≤10 mg/day within that period [12]. Gastrointestinal and hepatic toxicities are common during immunotherapy. Management of gastrointestinal irAEs relies on early recognition, prompt and adequate treatment, and rapid escalation of care [36]. For diarrhea (pooled incidence 14%), physicians should establish a baseline before treatment and regularly assess symptoms. New-onset or worsening diarrhea should be evaluated to exclude other causes such as infection, tumor progression, or concomitant medications [37]. Interestingly, a meta-analysis showed that irAEs are associated with clinical benefit, as patients developing irAEs had higher ORR, longer PFS, and longer OS, irrespective of ICI or irAE type [38]. These findings highlight the need for close monitoring and proactive management of irAEs, and clinicians should intervene promptly when adverse events occur. Treatment should be initiated early, even before diagnostic workup is complete, in patients with highly suggestive symptoms [33].

In subgroup analyses, the incidence of any-grade TRAEs was lower in the cadonilimab plus targeted therapy group than in the cadonilimab plus chemotherapy group, but both combination groups had high DCR (84% and 90%, respectively), both exceeding the monotherapy group’s DCR of 57%. These indirect subgroup comparisons are exploratory and require prospective confirmation. Additionally, the incidence of any-grade TRAEs was lower in retrospective studies than in single-arm trials and lower in single-center studies than in multicenter studies. These differences may be attributable to selection bias in retrospective and single-center studies, which may have enrolled patients with better baseline status. After subgroup analysis, heterogeneity was substantially reduced or eliminated in most subgroups, but moderate heterogeneity persisted in some subgroups. Residual heterogeneity may arise from several sources. First, variability across hospitals in management practices, data completeness, adverse event reporting thresholds, and follow-up may cause outcome fluctuations even within the same tumor type and regimen. Second, no uniform standards for drug exposure intensity or treatment duration exist across studies, and differences in dosing cycles, dose adjustments, and discontinuation criteria may introduce additional uncertainty. Variation in treatment lines is another potential contributor. Third, the recognition and management of irAEs improved progressively over the study period, potentially causing systematic shifts in reported data across publication years. Clinicians should therefore individualize treatment based on patient-specific factors and real-world evidence.

This study has several strengths. First, it included all available evidence from clinical trials and real-world studies published since 2023, reflecting recent advances in immunotherapy. Second, at the time of this systematic search, this represents the first meta-analysis to specifically evaluate the efficacy and safety of cadonilimab in digestive system neoplasms. Third, we analyzed a broad range of outcomes and performed extensive subgroup analyses to characterize the effects of cadonilimab from multiple perspectives. However, several limitations should be acknowledged. First, adverse event reporting was often incomplete: included studies typically reported only events exceeding a preset threshold (e.g., 10% or 15%), and rare or low-incidence events may have been omitted. This could lead to an incomplete safety assessment and affect the accuracy and clinical relevance of the conclusions. Second, as the application of cadonilimab in digestive system neoplasms is still in its infancy, current evidence is limited by small sample sizes and short follow-up periods, leaving certain critical survival data immature. This study refrained from pooling long-term endpoints (such as OS and PFS rates) and certain tumor response metrics, thereby limiting our ability to draw definitive conclusions regarding the drug’s long-term efficacy. Third, all included studies were conducted in China, which may limit the generalizability of our findings. Differences in disease etiology, biomarker prevalence, and treatment patterns between East Asian and Western populations could affect the applicability of the pooled results. Pooled efficacy metrics across biologically distinct tumors may not be directly comparable. Fourth, although sensitivity and subgroup analyses resolved heterogeneity for some outcomes, sources of heterogeneity remained unclear for others. High heterogeneity limits the reliability of pooled estimates, and readers should prioritize tumor-specific subgroup results in clinical interpretation. Fifth, this study employed the MINORS instrument for quality assessment. While suited to single-arm non-comparative designs, this assessment primarily evaluates reporting completeness rather than risk of bias directly. Sixth, because all pooled estimates derive from single-arm data and cannot be interpreted as comparative efficacy evidence, the limited quantity and quality of included studies further reinforce the need for cautious interpretation.

## 5. Conclusions

In conclusion, this study suggests that cadonilimab has the potential for antitumor activity in digestive system neoplasms, with a safety profile that merits close monitoring. However, conclusions drawn from the limited available studies must be interpreted with caution, and their long-term clinical efficacy requires further investigation. These results offer a descriptive summary of currently available data and should not be regarded as definitive evidence of efficacy. In clinical practice, careful management and monitoring of adverse events are essential. Additionally, the potential risks and benefits of combination regimens should be carefully assessed before treatment initiation.

## Figures and Tables

**Table 1:** Basic characteristics of included studies and literature quality evaluation results.

Study	Tumor Type ^b^	Study Identifier	Study Design	Sample Size	Median Age	Treatment Line	OS/PFS (Median)	Follow-Up (Median, Mouths)	PD-L1 Status	Treatment Protocol	Assessed Outcomes	CTCAE Version	Score
(Gao 2023, [15]) ^a^	ESCC/HCC	NCT03852251	Single-arm trial	22/24 ^c^	63/51 ^d^	prior lines ≤ 1	9.4/3.5; NR/3.7	17.9/19.6	CPS ≥ 1 (50%)/CPS ≥ 1 (21%)	Cadonilimab monotherapy	①②⑧	v5.0	14
(Qu 2025, [16])	ESCC	NCT05522894	Single-arm trial	43	61	1L	NR/7.1	12.88	CPS ≥ 1 (72.1%)	Cadonilimab plus chemotherapy	①④⑤⑥⑦⑧	v5.0	14
(Yuan 2024, [17])	HCC	NFEC-2021-044	Retrospective study	29	51.0 ± 11.8 ^f^	1L	NR/8.1	NR	NR	Cadonilimab plus Lenvatinib	①②③④⑥⑧	v5.0	10
(Qiao 2023, [18])	HCC	NCT04444167	Single-arm trial	59	56.3	1L	26.9/9.7	27.4	NR	Cadonilimab plus Lenvatinib	①②③④⑥⑧	v5.0	14
(Wang 2025, [19])	HCC	KY-2024-10-153-1	Retrospective study	78 ^e^	57.2 ± 11.1 ^f^	1L (53.8%), ≥2L (46.2%)	8.8/3.6	NR	NR	Cadonilimab plus TKI	①②③④⑥⑧	v5.0	17
(Shen 2025, [20])	G/GEJ adenocarcinoma	NCT05008783	Randomized controlled trial	305 ^g^	64 ^g^	1L	14.1/7.0	18.7	CPS ≥ 5 (38.0%)	Cadonilimab plus chemotherapy ^g^	①②③④⑤⑥⑦⑧	v5.0	7
(Gao, 2024, [21])	G/GEJ adenocarcinoma	CTR20182027	Single-arm trial	94	62.7	1L	17.48/8.18	29.2	CPS ≥ 1 (44.7%)	Cadonilimab plus chemotherapy	①②③④⑤⑥⑦⑧	v5.0	14
(Long 2025, [22])	G/GEJ adenocarcinoma	ChiCTR2200066893	Single-arm trial	38	57	1L	NR/NR	Not yet been reached	CPS ≥ 5 (34.2%)	Cadonilimab plus chemotherapy	①②③④⑥⑧	v5.0	12
(Liu 2025, [23])	G/GEJ cancer ^h^	YYYIRB-IIT [2025]014	Retrospective study	50 ^g^	—	2L	10.3/4.9	8.7	CPS ≥ 1 (56.0%)	Cadonilimab plus chemotherapy ^g^	①②③⑧	v4.0	17
(Qin 2025, [24])	PDAC	2025A-558	Retrospective study	14	56.5	NR	11.45/7.87	NR	NR	Cadonilimab plus chemotherapy	①②③④⑤⑥⑦⑧	v5.0	10

Assessed outcomes: ① DCR; ② PR; ③ PD; ④ any-grade TRAEs; ⑤ any-grade irAEs; ⑥ ≥3 grade TRAEs; ⑦ ≥3 grade irAEs; ⑧ specific categories of adverse events. ^a^. As the study (Gao-2023 [15]) comprised three tumor cohorts, only the digestive system tumors were evaluated in our meta-analysis, with the esophageal squamous cell cancer cohort defined as cohort A and the hepatocellular carcinoma cohort as cohort B. ^b^. Four included malignancies: ESCC, esophageal squamous cell carcinoma; HCC, hepatocellular carcinoma; G/GEJ adenocarcinoma, gastric/gastroesophageal junction adenocarcinoma; PDAC, pancreatic ductal adenocarcinoma. ^c^. Sample size: 22 in cohort A, 24 in cohort B. ^d^. Median age: 63 in cohort A, 51 in cohort B. ^e^. Only 32 of the 78 enrolled patients were evaluable for efficacy. ^f^: $\bar{x}\;$ ± s. ^g^. The arm receiving cadonilimab-based therapy. ^h^. Given that G/GEJ cancer is predominantly adenocarcinoma, it was merged with G/GEJ adenocarcinoma for the meta-analysis.

**Table 2:** Summary results of specific categories of adverse events.

Outcomes		No. of Arms *	Ratio (95% CI)	Heterogeneity	
				I^2^ (%)	*p*
Hematologic toxicity	Thrombocytopenia	11	29% (15–46%)	94.71	<0.01
	Neutropenia	11	42% (28–56%)	91.89	<0.01
	Leukocytopenia	10	43% (29–57%)	91.62	<0.01
	Anemia	9	35% (22–48%)	91.36	<0.01
Gastrointestinal toxicity	Decreased appetite	8	21% (11–33%)	87.94	<0.01
	Weight loss	8	21% (8–37%)	92.46	<0.01
	Diarrhea	8	14% (6–24%)	85.31	<0.01
	Nausea	7	48% (24–72%)	96.56	<0.01
Hepatic toxicity	AST elevation	9	33% (28–39%)	37.64	0.12
	ALT elevation	9	22% (15–30%)	73.24	<0.01
	Hyperbilirubinemia	7	20% (15–25%)	47.34	0.08
	Hypoalbuminemia	7	20% (10–32%)	86.46	<0.01

* Among them, the study by (Gao et al., 2023 [15]) included in this analysis analyzed two separate cohorts: an ESCC cohort (defined as cohort A) and an HCC cohort (defined as cohort B), thus resulting in a total of 11 arms.

**Table 3:** Summary results of subgroup analysis for DCR.

Subgroup (DCR)		No. of Arms	Ratio (95% CI)	Heterogeneity	
				I^2^ (%)	*p*
Tumor type	ESCC	2	87% (77–94%)	0.00	—
	HCC	4	79% (64–91%)	72.97	0.01
	PDAC	1	86% (57–98%)	—	—
	G/GEJ adenocarcinoma	4	88% (79–95%)	79.43	<0.01
Combination therapy	Cadonilimab monotherapy	2	57% (42–71%)	0.00	—
	Cadonilimab plus chemotherapy	6	90% (82–96%)	76.08	<0.01
	Cadonilimab plus targeted therapy	3	84% (70–94%)	65.54	0.05
Study design	Single-arm trial	6	86% (71–96%)	87.44	<0.01
	Retrospective study	4	77% (69–84%)	0.00	0.65
	Randomized controlled trial	1	87% (82–90%)	—	—
Research scale	Multicenter	7	87% (77–94%)	84.96	<0.01
	Single-center	4	77% (69–84%)	0.00	0.65

Note: All retrospective studies were single-center.

**Table 4:** Summary results of subgroup analysis for any-grade TRAEs.

Subgroup (Any-Grade TRAEs)		No. of Arms	Ratio (95% CI)	Heterogeneity	
				I^2^ (%)	*p*
Tumor type	ESCC	1	100% (92–100%)	—	—
	HCC	3	95% (79–100%)	87.61	<0.01
	PDAC	1	100% (77–100%)	—	—
	G/GEJ adenocarcinoma	3	99% (98–100%)	0.00	0.58
Combination therapy	Cadonilimab plus chemotherapy	5	100% (99–100%)	0.00	0.85
	Cadonilimab plus targeted therapy	3	95% (79–100%)	87.61	<0.01
Study design	Single-arm trial	4	100% (98–100%)	0.00	0.60
	Retrospective study	3	92% (82–99%)	53.36	0.12
	Randomized controlled trial	1	99% (97–100%)	—	—
Research scale	Multicenter	5	99% (98–100%)	0.00	0.76
	Single-center	3	92% (82–99%)	53.36	0.12

Note: All retrospective studies were single-center.

## Data Availability

No new data were created or analyzed in this study. Data sharing is not applicable to this article.

## Supplementary Materials

The following supporting information can be downloaded at https://www.mdpi.com/article/10.3390/ph19091348/s1, Supplementary File S1: The complete search strategies for all databases. Supplementary File S2: Quality assessment of included studies. Supplementary File S3: Figure S1: Forest plot results for PR. Figure S2: Forest plot results for PD. Figure S3: Forest plot results for ≥3 grade TRAEs. Figure S4: Forest plot results for ≥3 grade irAEs. Supplementary File S4: PRISMA-2020-checklist.

## Abbreviations

ESCC	esophageal squamous cell carcinoma
G/GEJ	gastric/gastroesophageal junction
HCC	hepatocellular carcinoma
PDAC	pancreatic ductal adenocarcinoma
DCR	disease control rate
PD	progressive disease
TRAEs	treatment-related adverse events
irAEs	immune-related adverse events
OS	overall survival
PFS	progression-free survival
CI	confidence interval
