# Supplementary material for: Efficacy and Safety of Cadonilimab in Digestive System Neoplasms: A Systematic Review and Single-Arm Meta-Analysis
Source: Pharmaceuticals (Basel). 2026 Aug 26;19(9):1348. doi: 10.3390/ph19091348 (PMC13610946; doi:10.3390/ph19091348)
Supplement: Supplementary file 1 [file pharmaceuticals-19-01348-s001.zip › Supplementary File S1.pdf]

Supplementary File S1: The complete search strategies for all databases.

PubMed		
#	Query	Results
#1	"Cadonilimab"[Title/Abstract] OR "AK104"[Title/Abstract] OR "PD-1/CTLA-4 bispecific"[Title/Abstract] OR "PD-1 CTLA-4 bispecific"[Title/Abstract] OR "anti-PD-1/CTLA-4"[Title/Abstract]	221
#2	"Digestive System Neoplasms"[MeSH Terms] OR "liver"[Title/Abstract] OR "hepatic"[Title/Abstract] OR "hepatocellular"[Title/Abstract] OR "stomach"[Title/Abstract] OR "gastric"[Title/Abstract] OR "esophageal"[Title/Abstract] OR "esophagus"[Title/Abstract] OR "pancreatic"[Title/Abstract] OR "pancreas"[Title/Abstract] OR "colorectal"[Title/Abstract] OR "colon"[Title/Abstract] OR "rectal"[Title/Abstract] OR "cholangiocarcinoma*"[Title/Abstract] OR "gallbladder"[Title/Abstract] OR "biliary"[Title/Abstract]	2,711,110
#3	"cancer*"[Title/Abstract] OR "tumor*"[Title/Abstract] OR "tumour*"[Title/Abstract] OR "neoplasm*"[Title/Abstract] OR "carcinoma*"[Title/Abstract]	4,435,988
#4	#1 AND #2 AND #3	73

Embase		
#	Query	Results
#1	'cadonilimab':ti,ab,kw OR 'ak104':ti,ab,kw OR 'pd-1/ctla-4 bispecific':ti,ab,kw OR 'pd-1 ctla-4 bispecific':ti,ab,kw OR 'anti-pd-1/ctla-4':ti,ab,kw	821
#2	'digestive system tumor'/exp	1,583,891
#3	'gastrointestinal tumor'/exp	878,808
#4	'liver':ti,ab,kw OR 'hepatic':ti,ab,kw OR 'hepatocellular':ti,ab,kw OR 'stomach':ti,ab,kw OR 'gastric':ti,ab,kw OR 'esophageal':ti,ab,kw OR 'esophagus':ti,ab,kw OR 'pancreatic':ti,ab,kw OR 'pancreas':ti,ab,kw OR 'colorectal':ti,ab,kw OR 'colon':ti,ab,kw OR 'rectal':ti,ab,kw OR 'cholangiocarcinoma*':ti,ab,kw OR 'gallbladder':ti,ab,kw OR 'biliary':ti,ab,kw	3,679,430

#5	#2 OR #3 OR #4	4,000,453
#6	'cancer*':ti,ab,kw OR 'tumor*':ti,ab,kw OR 'tumour*':ti,ab,kw OR 'neoplasm*':ti,ab,kw OR 'carcinoma*':ti,ab,kw	6,294,005
#7	#1 AND #5 AND #6	327

Cochrane Library		
#	Query	Results
#1	(‘Cadonilimab’ OR ‘AK104’ OR ‘PD-1/CTLA-4 bispecific’ OR ‘PD-1 CTLA-4 bispecific’ OR ‘anti-PD-1/CTLA-4’):ti,ab,kw	128
#2	MeSH descriptor: [Digestive System Neoplasms] explode all trees	29,031
#3	(‘liver’ OR ‘hepatic’ OR ‘hepatocellular’ OR ‘stomach’ OR ‘gastric’ OR ‘esophageal’ OR ‘esophagus’ OR ‘pancreatic’ OR ‘pancreas’ OR ‘colorectal’ OR ‘colon’ OR ‘rectal’ OR ‘cholangiocarcinoma*’ OR ‘gallbladder’ OR ‘biliary’):ti,ab,kw	200,939
#4	(‘cancer*’ OR ‘tumor*’ OR ‘tumour*’ OR ‘neoplasm*’ OR ‘carcinoma*’):ti,ab,kw	303,583
#5	#1 AND (#2 OR #3) AND #4	57

	Web of Science	
#	Query	Results
#1	“Cadonilimab” OR “AK104” OR “PD-1/CTLA-4 bispecific” OR “PD-1 CTLA-4 bispecific” OR “anti-PD-1/CTLA-4” (Topic)	388
#2	“Digestive System Neoplasms” OR “liver” OR “hepatic” OR “hepatocellular” OR “stomach” OR “gastric” OR “esophageal” OR “esophagus” OR “pancreatic” OR “pancreas” OR “colorectal” OR “colon” OR “rectal” OR “cholangiocarcinoma*” OR “gallbladder” OR “biliary” (Topic)	2,387,918
#3	“cancer*” OR “tumor*” OR “tumour*” OR “neoplasm*” OR “carcinoma*” (Topic)	4,749,477
#4	#1 AND #2 AND #3	132

	Wiley online library	
#	Query	Results
#1	“Cadonilimab” OR “AK104” OR “PD-1/CTLA-4 bispecific” OR “PD-1 CTLA-4 bispecific” OR “anti-PD-1/CTLA-4” in Abstract	11
#2	“Digestive System Neoplasms” OR “liver” OR “hepatic” OR “hepatocellular” OR “stomach” OR “gastric” OR “esophageal” OR “esophagus” OR “pancreatic” OR “pancreas” OR “colorectal” OR “colon” OR “rectal” OR “cholangiocarcinoma*” OR “gallbladder” OR “biliary”	315,026
#3	“cancer*” OR “tumor*” OR “tumour*” OR “neoplasm*” OR “carcinoma*”	480,694
#4	#1 AND #2 AND #3	4

CNKI	
Query	Results
(TKA='卡度尼利单抗' OR TKA='AK104' OR TKA='抗 PD-1/CTLA-4' OR TKA='PD-1/CTLA-4 双特异性') AND (TKA='结直肠癌' OR TKA='食管癌' OR TKA='胃癌' OR TKA='肝细胞癌' OR TKA='胰腺癌' OR TKA='胆管癌' OR TKA='消化系统肿瘤')	13

VIP	
Query	Results
(T=卡度尼利单抗 OR T=AK104 OR T="抗 PD-1/CTLA-4" OR T="PD-1/CTLA-4 双特异性") AND (T=结直肠癌 OR T=食管癌 OR T=胃癌 OR T=肝细胞癌 OR T=胰腺癌 OR T=胆管癌 OR T=消化系统肿瘤)	5

Wanfang	
Query	Results
主题:(卡度尼利单抗 OR AK104 OR "抗 PD-1/CTLA-4" OR "PD-1/CTLA-4 双特异性") AND 主题:(结直肠癌 OR 食管癌 OR 胃癌 OR 肝细胞癌 OR 胰腺癌 OR 胆管癌 OR 消化系统肿瘤)	16

CBM	
Query	Results
("卡度尼利单抗"[标题:智能] OR "AK104"[标题:智能] OR "抗 PD-1/CTLA-4"[标题:智能] OR "PD-1/CTLA-4 双特异性"[标题:智能]) AND ("结直肠癌"[常用字段:智能] OR "食管癌"[常用字段:智能] OR "胃癌"[常用字段:智能] OR "肝细胞癌"[常用字段:智能] OR "胰腺癌"[常用字段:智能] OR "胆管癌"[常用字段:智能] OR "消化系统肿瘤"[常用字段:智能])	5
