# Supplementary material for: Efficacy and Safety of Cadonilimab in Digestive System Neoplasms: A Systematic Review and Single-Arm Meta-Analysis
Source: Pharmaceuticals (Basel). 2026 Aug 26;19(9):1348. doi: 10.3390/ph19091348 (PMC13610946; doi:10.3390/ph19091348)
Supplement: Supplementary file 1 [file pharmaceuticals-19-01348-s001.zip › Supplementary File S2.pdf]

Supplementary File S2: Quality assessment of included studies.

Table S1. Quality assessment of non-randomized studies using the MINORS instrument.

Item	1	2	3	4	5	6	7	8	9	10	11	12	Total
Gao 2023 [15]	2	2	2	2	1	2	1	2	—	—	—	—	14
Qu 2025 [16]	2	2	2	2	1	2	1	2	—	—	—	—	14
Yuan 2024 [17]	2	2	2	2	1	1	0	0	—	—	—	—	10
Qiao 2023 [18]	2	2	2	2	1	2	1	2	—	—	—	—	14
Wang 2025 [19]	2	2	1	2	1	1	1	0	2	1	2	2	17
Gao 2024 [21]	2	2	2	2	1	2	1	2	—	—	—	—	14
Long 2025 [22]	2	2	2	2	1	1	0	2	—	—	—	—	12
Liu 2025 [23]	2	2	1	2	1	2	0	0	2	1	2	2	17
Qin 2025 [24]	2	2	1	2	1	2	0	0	—	—	—	—	10

Details:

1. A clearly stated aim.
2. Inclusion of consecutive patients.
3. Prospective collection of data
4. Endpoints appropriate to the aim of the study.
5. Unbiased assessment of the study endpoint.
6. Follow-up period appropriate to the aim of the study.
7. Loss to follow-up of less than 5%.
8. Prospective calculation of the study size.
9. An adequate control group.
10. Contemporary groups.
11. Baseline equivalence of groups.
12. Adequate statistical analyses.

The items were scored as 0 (not reported), 1 (reported but inadequate), or 2 (reported and adequate). The global ideal score was 16 for noncomparative studies and 24 for comparative studies.

Table S2. Quality assessment of randomized controlled trials using the Jadad scale.

Item	Randomization sequence generation	Allocation concealment	Blinding	Withdrawals and drops	Total
Shen 2025 [20]	2	2	2	1	7

Details (Shen 2025):

1.Randomization sequence generation: using an interactive web response system by the stratified block randomization approach

2.Allocation concealment: using an interactive web response system

3.Blinding: double-blind design

4.Withdrawals and drops: adequately reported
