# Supplementary material for: Efficacy and Safety of Cadonilimab in Digestive System Neoplasms: A Systematic Review and Single-Arm Meta-Analysis
Source: Pharmaceuticals (Basel). 2026 Aug 26;19(9):1348. doi: 10.3390/ph19091348 (PMC13610946; doi:10.3390/ph19091348)
Supplement: Supplementary file 1 [file pharmaceuticals-19-01348-s001.zip › Supplementary File S3.pdf]

Supplementary File S3: Figure S1: Forest plot results for PR. Figure S2: Forest plot results for PD. Figure S3: Forest plot results for ≥3 grade TRAEs. Figure S4: Forest plot results for ≥3 grade irAEs.

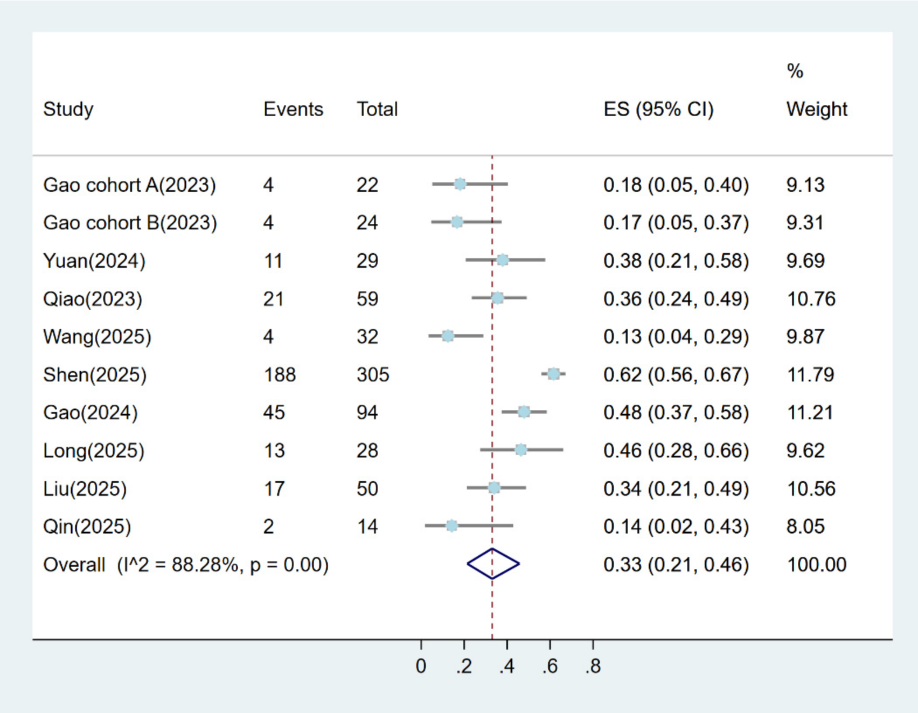

Figure S1. Forest plot results for PR.

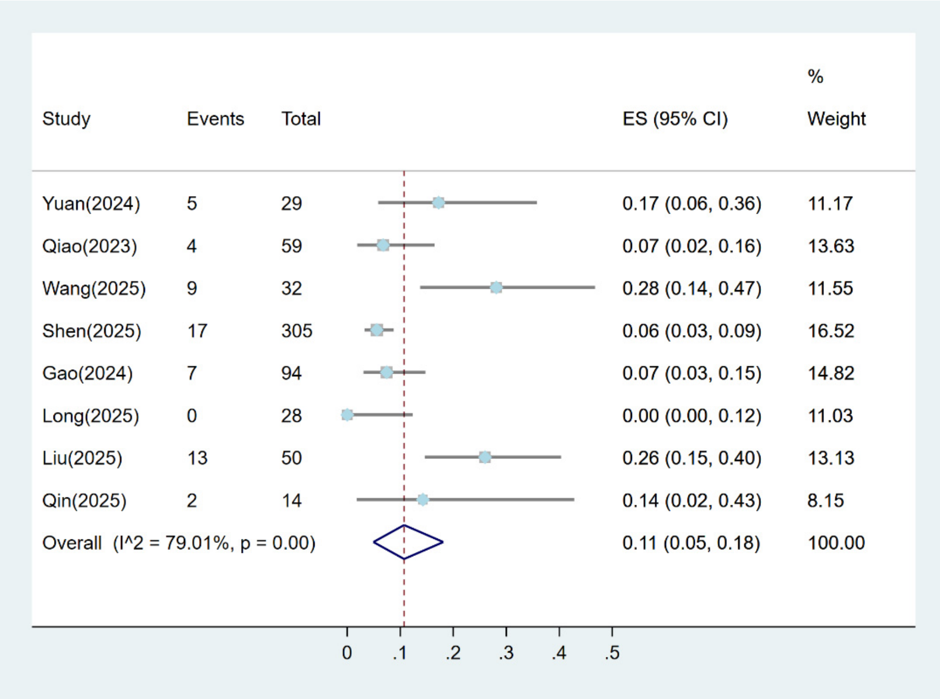

Figure S2. Forest plot results for PD.

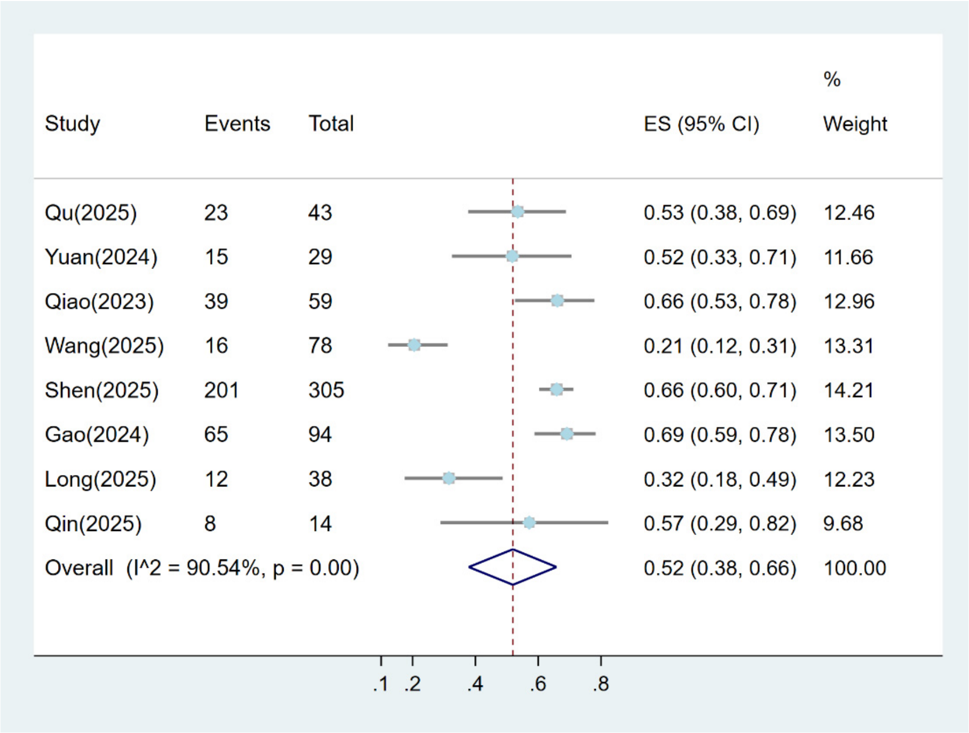

Figure S3. Forest plot results for ≥3 grade TRAEs.

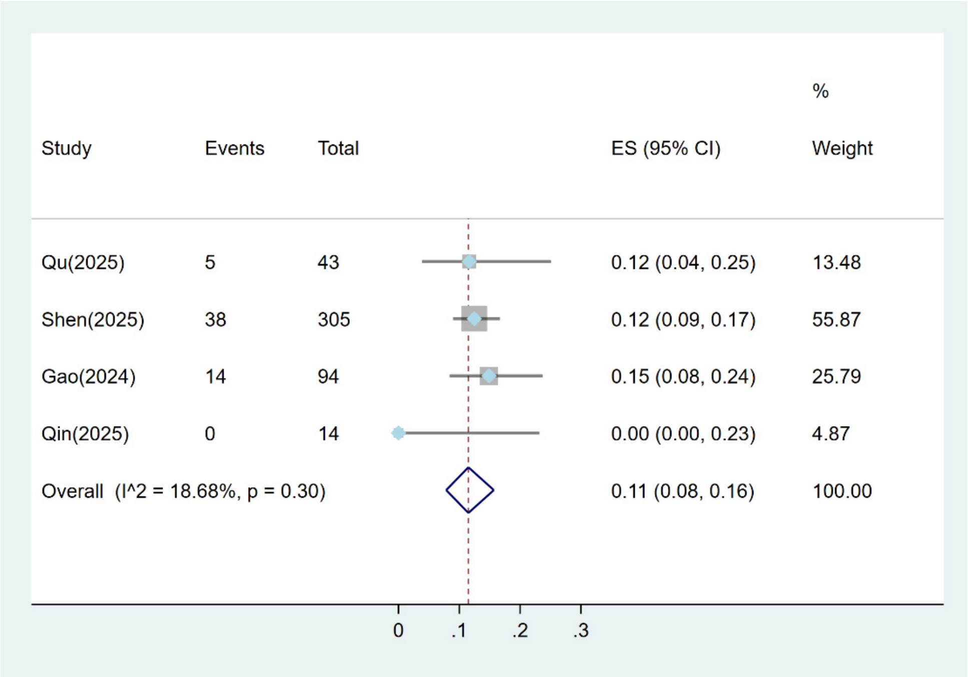

Figure S4. Forest plot results for ≥3 grade irAEs.
